# Improving diagnostic precision in amyloid brain PET imaging through data-driven motion correction

**DOI:** 10.1186/s40658-024-00653-z

**Published:** 2024-06-14

**Authors:** Hye Lim Park, Sonya Youngju Park, Mingeon Kim, Soyeon Paeng, Eun Jeong Min, Inki Hong, Judson Jones, Eun Ji Han

**Affiliations:** 1 Division of Nuclear Medicine, Department of Radiology, Eunpyeong St. Mary’s Hospital, College of Medicine, The Catholic University of Korea, Seoul, Republic of Korea; 2 Division of Nuclear Medicine, Department of Radiology, Yeouido St. Mary’s Hospital, College of Medicine, The Catholic University of Korea, 10, 63-ro, Yeongdeungpo-gu, Seoul, 07345 Republic of Korea; 3 Siemens Healthineers Ltd, Seoul, Republic of Korea; 4 Department of Biomedicine & Health Sciences, The Catholic University of Korea, Seoul, Republic of Korea; 5 Department of Medical Life Sciences, College of Medicine, The Catholic University of Korea, Seoul, Republic of Korea; 6 Siemens Medical Solutions USA, Inc., Knoxville, TN USA

**Keywords:** Amyloid, Motion correction, ^18^F-flutemetamol, PET/CT, Brain

## Abstract

**Background:**

Head motion during brain positron emission tomography (PET)/computed tomography (CT) imaging degrades image quality, resulting in reduced reading accuracy. We evaluated the performance of a head motion correction algorithm using ^18^F-flutemetamol (FMM) brain PET/CT images.

**Methods:**

FMM brain PET/CT images were retrospectively included, and PET images were reconstructed using a motion correction algorithm: (1) motion estimation through 3D time-domain signal analysis, signal smoothing, and calculation of motion-free intervals using a Merging Adjacent Clustering method; (2) estimation of 3D motion transformations using the Summing Tree Structural algorithm; and (3) calculation of the final motion-corrected images using the 3D motion transformations during the iterative reconstruction process. All conventional and motion-corrected PET images were visually reviewed by two readers. Image quality was evaluated using a 3-point scale, and the presence of amyloid deposition was interpreted as negative, positive, or equivocal. For quantitative analysis, we calculated the uptake ratio (UR) of 5 specific brain regions, with the cerebellar cortex as a reference region. The results of the conventional and motion-corrected PET images were statistically compared.

**Results:**

In total, 108 sets of FMM brain PET images from 108 patients (34 men and 74 women; median age, 78 years) were included. After motion correction, image quality significantly improved (*p* < 0.001), and there were no images of poor quality. In the visual analysis of amyloid deposition, higher interobserver agreements were observed in motion-corrected PET images for all specific regions. In the quantitative analysis, the UR difference between the conventional and motion-corrected PET images was significantly higher in the group with head motion than in the group without head motion (*p* = 0.016).

**Conclusions:**

The motion correction algorithm provided better image quality and higher interobserver agreement. Therefore, we suggest that this algorithm be adopted as a routine post-processing protocol in amyloid brain PET/CT imaging and applied to brain PET scans with other radiotracers.

## Introduction

Alzheimer’s disease (AD) is the leading cause of dementia, accounting for 60–70% of all dementia cases [1]. Furthermore, the number of patients with dementia is expected to significantly increase owing to aging and population growth trends. AD represents a major medical challenge worldwide and carries a tremendous socio-economic burden [2]. The root cause of AD remains unknown; however, extracellular amyloid-beta (Aβ) deposition in the brain is the predominant hypothesis [3]. The two most common clinical diagnostic criteria for AD are the Diagnostic and Statistical Manual of Mental Disorders (5th edition) and the National Institute on Aging-Alzheimer’s Association (NIA-AA) [4]. The NIA-AA criteria consist of amyloid (A), tau (T), and neurodegeneration (N). For amyloid evaluation, cerebrospinal fluid analysis or amyloid brain positron emission tomography (PET) scan is recommended [5].

Amyloid brain PET has enabled non-invasive assessment of Aβ deposition as a molecular biomarker since its introduction in the early 2000s. Amyloid PET can support the evaluation of those with cognitive impairment and the diagnosis of AD through early detection of Aβ deposition and visualization of its distribution [6]. ^18^F-flutemetamol (FMM), a thioflavin derivative of Pittsburgh compound B, is one of the most widely used amyloid PET tracers [7]. Clinical physicians use visual binary analysis, either Aβ negative or Aβ positive, on amyloid PET. To date, quantitative analysis using standard uptake value ratio (SUVR) has been mainly used in research settings. However, as new drugs for AD will soon be used in clinical practice, quantitative evaluation has the potential to become an essential tool for monitoring disease progression and treatment responses [8].

It is a well-known problem that PET images are blurred and quantitatively inaccurate owing to periodic movements such as cardiac or respiratory motion. To overcome this challenge, data-driven methods to correct cardiac or respiratory motion have been commercialized and implemented for cardiac and whole-body PET imaging [9, 10]. However, data-driven methods for brain PET imaging are still under investigation. For amyloid brain PET scans, it is recommended to obtain static images for 10–20 min [11]. However, patients suspected of neurodegenerative diseases tend to be older and do not tolerate a 20-min data acquisition without moving. Some patients even make small movements imperceptible to human observation. These head movements degrade PET image quality, leading to decreased reading accuracy and inaccurate quantification [12].

This study aimed to evaluate the performance of a data-driven head motion correction technique for FMM brain PET imaging. The consistency of the visual assessment was analyzed using PET images before and after head motion correction, and quantitative values were compared.

## Methods

### Patients

We retrospectively collected FMM PET/computed tomography (CT) scans from patients with memory impairment who visited our hospital between December 2021 and August 2022. This study was approved by the Institutional Review Board of our institution (IRB no. SC22RISI0123). The requirement for informed consent was waived because of its retrospective design. This study was conducted in accordance with the relevant guidelines and regulations of the ethics committee.

### FMM PET/CT acquisition

All PET/CT studies were performed using a PET/CT scanner (Biograph Vision 600, Siemens Healthineers, Hoffman Estates, IL). Patients received an intravenous injection of 185 MBq FMM, and scanning began approximately 90 min post-injection. CT was performed for attenuation correction, followed immediately by static PET for 20 min. PET images were reconstructed using 3-dimensional (3D) ordered-subset expectation maximization with time-of-flight (8 iterations, 5 subsets, matrix size 440 × 440, and voxel size 0.82 × 0.82 × 1.64 mm). No point spread function modelling or post-filtering was performed.

### Data-driven motion correction and image reconstruction

**Fig. 1:**
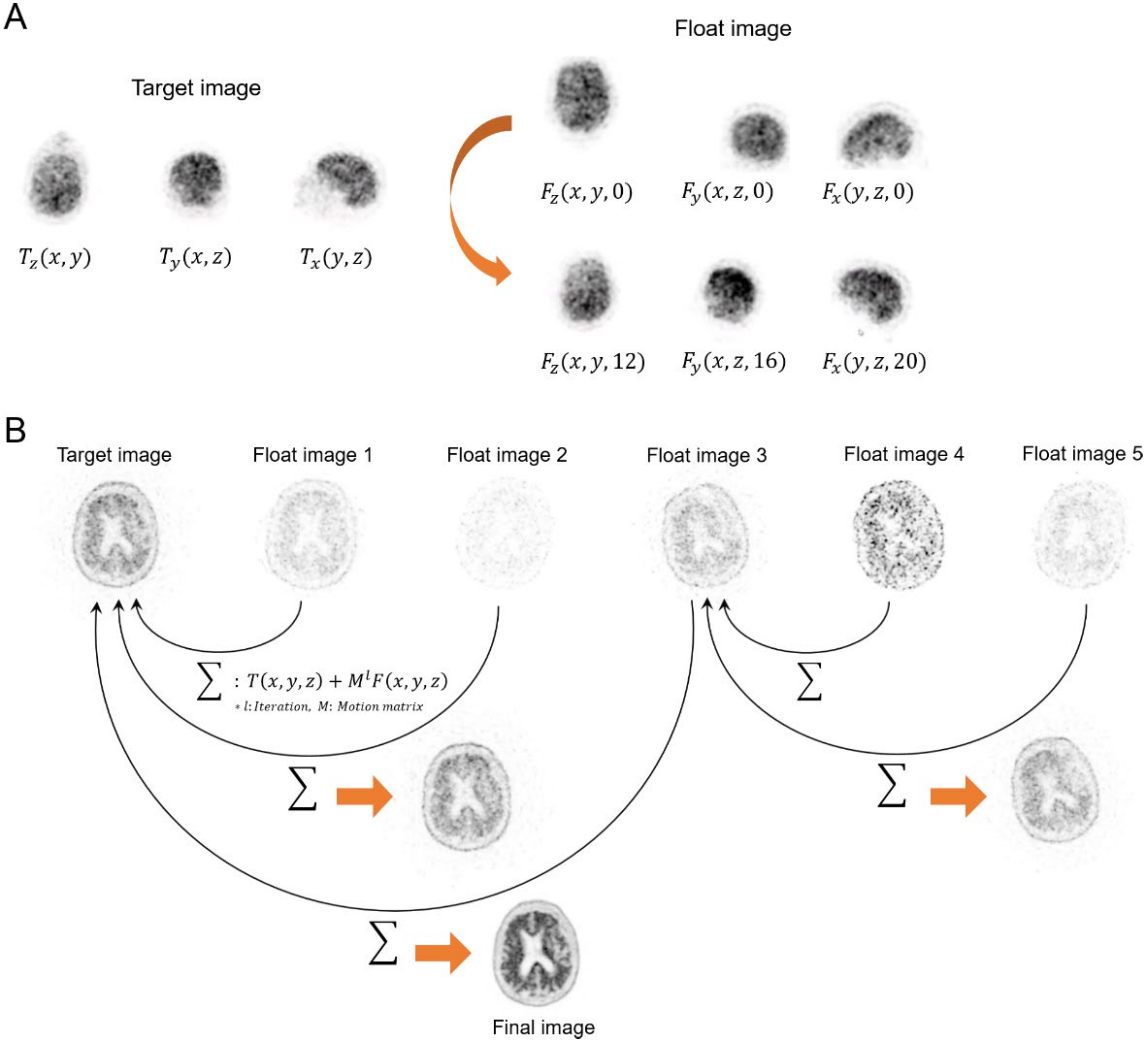
The summing tree structure method. (**A**) The iterative process of motion correction was performed from the floating image to the target image. (**B**) The summed target image became the floating image for a new tree node target image, and then, all the corrected motion frames were finally merged into a single frame

Data-driven motion correction was performed using an investigational software prototype (Siemens Healthineers, Knoxville, TN, USA) and involved the following three steps. First, we estimated the center of signal distribution in the X, Y, and Z dimensions at 1.0-s time intervals. This yielded a 3D time-domain signal at 1.0 Hz spanning the entire duration of the list-mode file. The signal was smoothed using an adaptive bilateral filter to reduce noise. Subsequently, the signal was divided into quiescent intervals without detectable motion, separated by motion events. The number of motion-free intervals was calculated using a previously developed clustering method that identified time-continuous intervals, without prior knowledge of the number of clusters [13]. Second, to estimate 3D motion transformations between the various intervals, we used the Summing Tree Structure algorithm (Fig. 1) [14]. We reconstructed the list-mode files in each interval without attenuation correction. The 3D images were projected into two 2D images. Using these two 2D images, a mutual information method estimated 3D transformations from a “floating” image to a “target” image according to the plan. The 3D transformations were applied to the floating 3D images. The transformed 3D images were again projected to two 2D images, and the process was repeated. When the iterations were complete, the 3D transformations represented how the floating images were motion corrected to the frames of the target images. Third, we used the 3D transformations to calculate a final motion-corrected image. There were several possible choices for the method in this step: first, motion correction in image space; second, motion correction in sinogram space; and third, motion correction during the iterative reconstruction process. We adopted the third method as it yielded the best results with respect to spatial resolution [15].

### PET image analysis

All conventional and motion-corrected FMM brain PET images were visually reviewed by two nuclear medicine physicians. The readers had completed the electronic training program provided by the manufacturer (http://www.vizamyl.co.kr) [16], and both had over 7 years of experience in assessing > 1,000 brain amyloid PET/CT images. The readers were blinded to all clinical information of the patients. For visual assessment, a spectrum color scale for the PET images was set to 90% of the pons signal intensity, and transverse, sagittal, and coronal views were displayed using commercial imaging software (syngo.via version VB60A; Siemens Healthineers). PET images were interpreted as negative, positive, or equivocal by comparing activity in cortical gray matter with activity in the adjacent white matter in 5 specific regions: the frontal lobe, parietal lobe, lateral temporal lobe, posterior cingulate/precuneus (PC/P), and striatum. If any of these regions were clearly positive, then the entire image was classified as positive. If all 5 regions were clearly negative, the entire image was classified as negative. PET image quality was evaluated using a 3-point scale: good, acceptable, and poor. Readers scored subjectively based on the prevalence of noise, contrast between different brain tissues (differentiation between the scalp, skull, cortex, and ventricles), and suitability of interpretation. For quantitative analysis, syngo.via MI Neurology (Siemens Healthineers) was used. After spatial normalization using the automated anatomical labeling atlas and a volume of interest (VOI) was set, the uptake ratio (UR) of each VOI was calculated semi-automatically using the cerebellar cortex as the reference region.

### Statistical analysis

Categorical variables were expressed as absolute numbers and percentages; continuous variables were expressed as medians and ranges. Visual inspection of image quality before and after motion correction was performed using the McNemar-Bowker test. Concordance among the two readers was evaluated by Cohen’s kappa (κ) statistics. A κ value of 0.0–0.2 was considered to represent slight agreement; 0.21–0.4, fair; 0.41–0.6, moderate; 0.61–0.8, substantial; and 0.81–1.0, almost perfect. We employed the Benjamini-Hochberg procedure to control the false discovery rate at a rate of 0.05 for multiple hypotheses testing. For quantitative analysis, the difference of UR between the conventional and motion-corrected images was evaluated using a t-test and the Kruskal-Wallis test. A *p* value less than 0.05 was considered statistically significant. All statistical analyses were performed using R (version 4.2.2, R Foundation for Statistical Computing, Vienna, Austria).

## Results

**Fig. 2:**
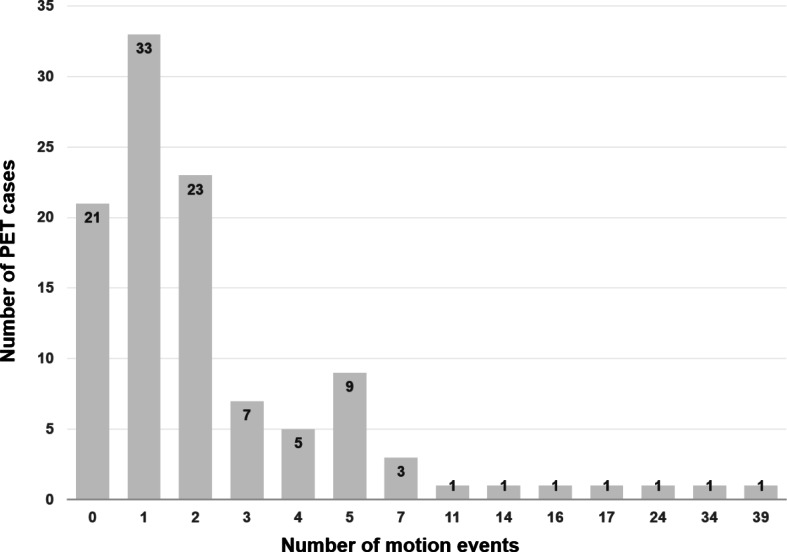
Number of head motion events detected by the motion correction algorithm in the 108 PET image sets

A total of 108 sets of FMM brain PET images from 108 patients (34 men and 74 women; median age, 78 years, range 52–92 years) were included in this study. Most patients (*n* = 99) were 65 years or older. For image quality, approximately three-quarters of the 108 conventional PET images were deemed to be of good quality on visual evaluation by each of the two readers (Table 1). Both readers unanimously agreed on the poor quality of the 5 PET images (5%). After motion correction, the image quality was significantly improved for both readers (*p* < 0.001), and there were no images of poor quality. Based on the number of events detected by the motion correction algorithm, absence of motion was seen in only 21 (19%) of the 108 patients. Of the remaining 87 patients, 56 (52%) had 1 or 2 motion events, 24 (22%) patients had 3 to 9 events, and 7 (7%) patients had ≥ 10 events (Fig. 2).

**Table 1:** Visual assessment of PET image quality by the two readers

	Conventional images (*n* = 108)		Motion-corrected images (*n* = 108)	
Image quality	Reader A	Reader B	Reader A	Reader B
Good	78 (72%)	82 (76%)	103 (95%)	105 (97%)
Acceptable	25 (23%)	21 (19%)	5 (5%)	3 (3%)
Poor	5 (5%)	5 (5%)	0 (0%)	0 (0%)

Of the 56 patients with 1 or 2 motion events, 41 (73%) patients were visually judged by both readers to have good image quality. In the visual assessment of amyloid deposition, reader A interpreted 45 (42%) of the 108 conventional PET images as amyloid positive and reader B interpreted 51 (47%) images as amyloid positive. Of the 108 motion-corrected PET images, readers A and B interpreted 47 (43%) and 48 (44%) as amyloid positive, respectively (Table 2). There were disagreements between the two readers in 11 (10%) of the conventional PET images, with most of these (*n* = 10/11) having head motion detected using the motion correction algorithm. The number of concordant cases increased from 97 to 101 after motion correction; however, the difference was not significant (*p* = 0.23). For the regional analysis, a higher interobserver agreement was observed in the motion-corrected PET images for all 10 specific regions, with a statistically significant difference in the left PC/P region (adjusted *p* = 0.038) (Table 3).

**Table 2:** PET interpretation for amyloid deposition by the two readers

	Conventional images (*n* = 108)		Motion-corrected images (*n* = 108)	
Interpretation	Reader A	Reader B	Reader A	Reader B
Amyloid positive	45 (42%)	51 (47%)	47 (43%)	48 (44%)
Amyloid equivocal	5 (4%)	5 (4%)	4 (4%)	4 (4%)
Amyloid negative	58 (54%)	52 (48%)	57 (53%)	56 (52%)

**Table 3:** Interobserver agreement by specific region

Region	Conventional images κ (95% CI)	Motion-corrected images κ (95% CI)
Frontal, right	0.84 (0.74–0.93)	0.89 (0.80–0.97)
Frontal, left	0.82 (0.72–0.92)	0.89 (0.80–0.97)
Parietal, right	0.92 (0.84–1.00)	0.92 (0.85–1.00)
Parietal, left	0.90 (0.82–0.98)	0.94 (0.88–1.00)
Temporal, right	0.81 (0.71–0.92)	0.92 (0.85–1.00)
Temporal, left	0.81 (0.71–0.92)	0.87 (0.78–0.96)
Striatum, right	0.72 (0.59–0.85)	0.85 (0.74–0.95)
Striatum, left	0.67 (0.54–0.80)	0.87 (0.77–0.97)
PC/P, right	0.77 (0.66–0.89)	0.92 (0.85–1.00)
PC/P, left	0.78 (0.67–0.90)	0.98 (0.94–1.00)

CI, confidence interval; PC/P, posterior cingulate/precuneus

Of the 108 PET image sets, 13 image sets could not be quantitatively assessed because of technical errors. Of the remaining 95 sets from 95 patients (34 men and 61 women; median age, 78 years, range 52–92 years), UR was significantly different between the conventional and motion-corrected PET images (*p* < 0.001). In the 18 sets (19%) without head motion, the median UR difference between the conventional and motion-corrected PET images was 0.06 (range, 0.01–0.22). In contrast, in the remaining 77 sets (81%) with head motion, the median UR difference was 0.10 (range, -0.15–0.77), which was significantly higher than that in the pairs without head motion (*p* = 0.016).

## Discussion

We evaluated the clinical performance of a recently developed head motion correction algorithm for amyloid brain PET images. Image quality and interobserver agreement significantly improved after motion correction. The difference in UR between the conventional and motion-corrected PET images was significantly greater in the group with head motion than in the group without head motion.

Unlike the lung, heart, or bowel, the brain exhibits negligible autonomous movement. However, amyloid brain PET requires acquisition times of typically 10–20 min, and patients undergoing amyloid PET are suspected to have dementia and are often unable to tolerate the study. Therefore, there is a high likelihood that subtle to large movements occur during PET scans. The current diagnostic criteria for amyloid PET include visual assessment of tracer uptake in the gray matter [11]; however, head motion makes it difficult to distinguish between the gray and white matter. Before the respiratory motion correction algorithm was developed for oncology whole-body PET, if the accurate evaluation of lesions in the lung base or upper abdomen was difficult owing to respiratory motion, the relevant area was imaged again. Head motion is unpredictable and prevalent in patients with poor motor control. In addition, the re-scan time would not be shorter than the initial scan time in amyloid brain PET, and the additional radiation exposure from the re-scan must be considered. Therefore, a post-acquisition head motion correction algorithm is necessary.

In this study, we applied an advanced motion correction algorithm to amyloid brain PET images and compared it to conventional methods. When dividing the list-mode file into a certain number of contiguous intervals according to specific criteria, we initially applied traditional K-means clustering to find intervals when the patient was stationary. However, the traditional algorithm was limited by requiring knowledge of the number of clusters in advance and by assigning observations to clusters regardless of their observation time. To overcome these limitations, we developed a modified “Merging Adjacent Clustering” method so that time-continuous intervals could be identified. Therefore, it could calculate the number of clusters without knowing in advance how many there are. In the process of estimation of 3D motion transformations among the various intervals, when the iterations are complete, the final image represents the final floating image, motion corrected to the frame of the reference image. For summing all corrected motion frames into one frame, rather than the typical method of registering all floating images to a single target, we applied a new “Summing Tree Structural Motion Correction” algorithm. This approach could reduce image noise effectively by summing the tree nodes of each image after motion correction. In addition, during the iterative reconstruction process, we calculated a motion correction method, resulting in a final image with spatial resolution nearly identical to that of the reference image acquired without motion.

The visual reading system for amyloid brain PET images used in routine clinical practice is intuitive and easy to use without any special program [11]. In previous studies, interobserver agreement for visual analysis has been reported to be good in well-trained readers (κ = 0.70–0.93) [17, 18]. In this study, there was almost perfect agreement between two nuclear medicine physicians with respect to interobserver assessment in the conventional and motion-corrected PET images (κ = 0.81 and κ = 0.88, respectively). In the regional analysis, higher interobserver agreement was observed in motion-corrected PET images in all specific regions, and there was a statistically significant increase in the left PC/P region (*p* = 0.038). The PC/P region is one of the earliest brain regions of the brain to be affected by Aβ pathology [19, 20], and it is associated with executive function changes that may precede memory decline in preclinical AD [20]. This region is relatively small compared to the frontal, parietal, and lateral temporal cortices in the transaxial plane and would thus be expected to be more affected by head motion. The increase in interobserver agreement in this area on motion-corrected PET images indicates that this algorithm may help improve the diagnostic performance of amyloid brain PET.

**Fig. 3:**
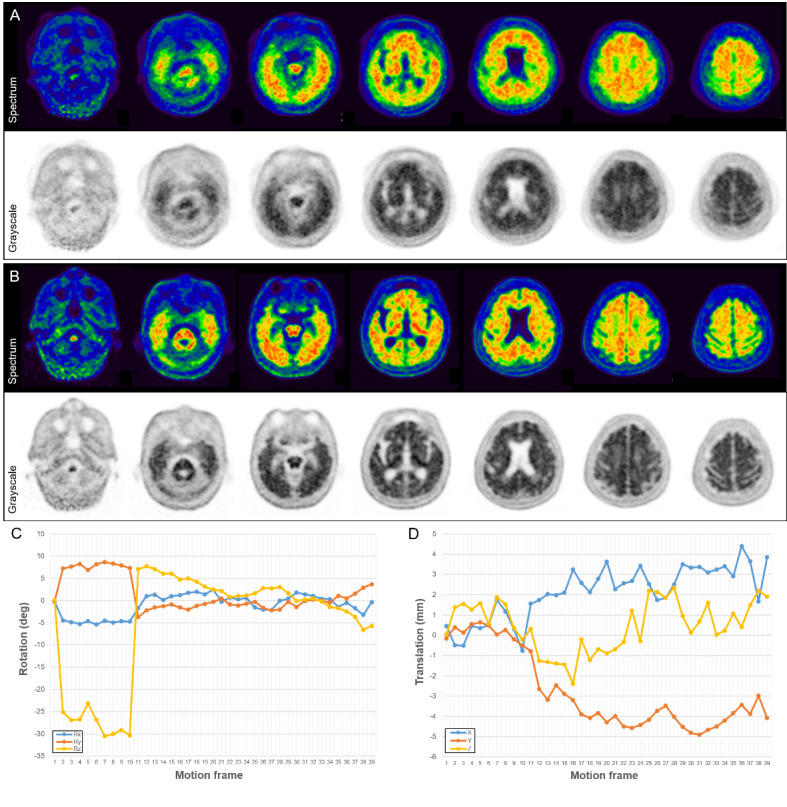
^18^F-flutemetamol brain PET study in a 92-year-old woman. Both readers interpreted the conventional PET images (**A**) as amyloid positive with poor image quality. The motion-corrected PET images (**B**) had good image quality and were still interpreted as amyloid positive. The graphs showed the rotation (**C**) and translation (**D**) estimated by the motion correction algorithm that revealed 39 frequent and large head movements

**Fig. 4:**
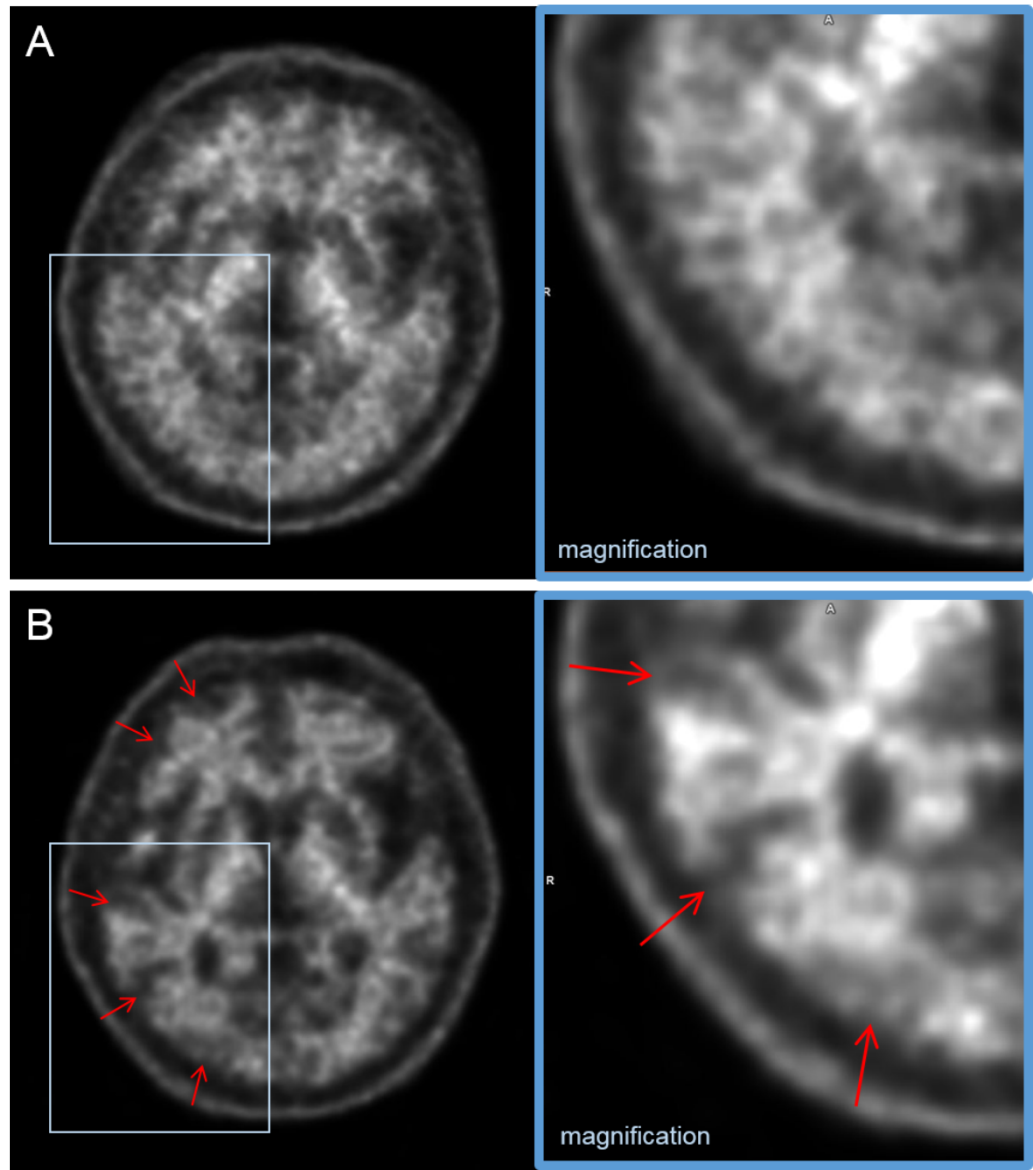
^18^F-flutemetamol brain PET images from a 67-year-old man with obvious head movements during scanning. Both readers interpreted the conventional PET images (**A**) as amyloid equivocal with poor image quality. After motion correction, the preserved gray matter to white matter contrast was clearly revealed on the PET images (**B**, arrows). Accordingly, both readers changed their interpretation to amyloid negative with good image quality

In this study, the final PET interpretations of amyloid deposition changed after motion correction in 11 (10%) of 108 PET image sets. However, the number of equivocal PET results did not change significantly before or after motion correction for either reader (*n* = 5 ◊ 5 for reader A; *n* = 4 ◊ 4 for reader B). Amyloid-equivocal results are inevitable in the binary visual assessment of amyloid brain PET images because of anatomical problems, such as severe atrophy, cortical deformity, or encephalomalacia, as well as the low burden of amyloid deposition [21]. However, the amyloid-equivocal results for head motion are expected to improve with the application of the motion correction algorithm. Of the 5 patients (5%) with poor image quality in the conventional PET images, 4 patients showed definite FMM uptake throughout the entire cerebral cortex; therefore, both readers judged them to have amyloid positivity despite severe motion, and these results did not change even after motion correction (Fig. 3). However, the remaining patient was judged to have amyloid equivocality owing to ambiguous uptake in some areas, and the final report of both readers was changed to amyloid negativity after motion correction (Fig. 4).

Quantitative assessment is widely used in ^18^F-fluorodeoxyglucose (FDG) PET for the staging and evaluation of treatment response in solid tumors. It shows excellent intra- and interobserver reproducibility and can provide more objective and detailed information than visual analysis [22–24]. For the evaluation of amyloid brain PET images in patients with cognitive impairment, quantitative assessment is mainly used in research settings [25]. Cholinesterase inhibitors are currently used to treat AD; however, they are not curative and cannot stop disease progression. Recently, monoclonal antibody drugs (e.g., lecanemab or donanemab) that can reduce Aβ accumulation have been actively developed, and accordingly, the role of amyloid and tau PET for quantifying Aβ and tau burden in vivo has been emphasized [26–28]. SUVR is a representative value for the quantification of amyloid and tau PET images and is widely used in clinical trials to evaluate the therapeutic effects of new drugs. Although the appropriate reference region and cut-off values for each radiotracer remain controversial, SUVR can be a supplementary parameter for clearer judgments in equivocal PET cases using a visual reading system [8, 29–31]. The accurate setting of the VOI along the gray matter is essential for obtaining accurate SUVR from amyloid brain PET. Unlike in oncology PET where the boundary between FDG uptake by the tumor and the background is clear, in amyloid brain PET, the boundary between the gray and white matter can be easily obscured, even by fine motion.

**Fig. 5:**
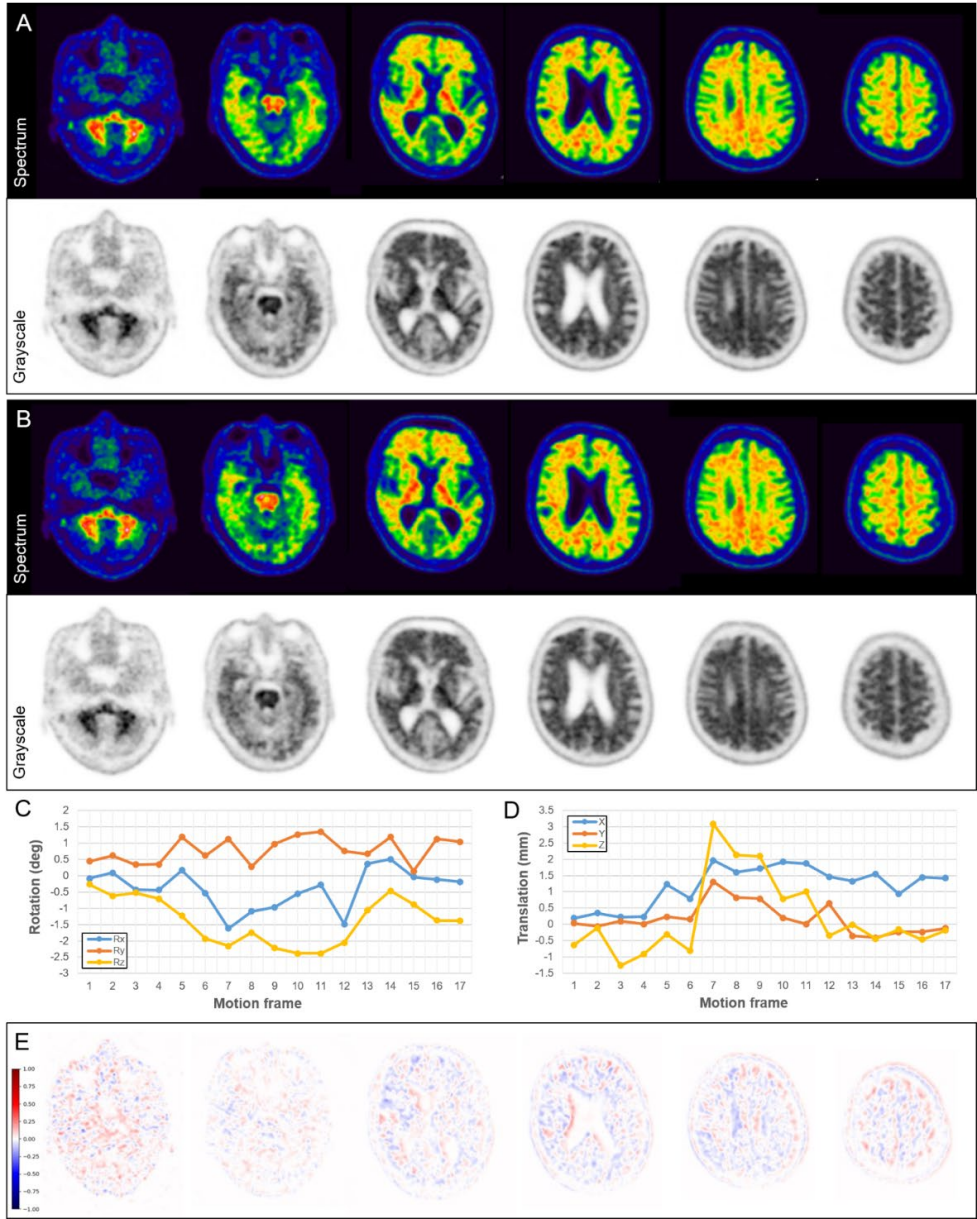
^18^F-flutemetamol brain PET study in a 79-year-old man. Both readers interpreted both conventional (**A**) and motion-corrected (**B**) PET images as amyloid positive with good image quality. However, the motion correction algorithm detected 17 subtle head movements with rotation changes of up to 2.39° (**C**) and translations of up to 3.09 mm (**D**) relative to the initial position. In quantitative analysis, the UR differed by 0.12 between the conventional and motion-corrected PET images. The difference in normalized SUV between both PET images was identified in the difference map by subtracting the conventional from the motion-corrected PET images (**E**)

Given that the uptake of white matter is much higher than that of gray matter in patients with amyloid-negative PET findings, improper inclusion of white matter uptake into the VOI due to head motion leads to a false elevation of the SUVR. In this study, 41 (73%) of the 56 conventional PET images with 1 or 2 motion events were visually assessed by both readers as having good image quality. Of the 68 conventional PET images assessed by both readers as having good image quality, 51 images had more than 1 motion event, including 1 image with as many as 17 motion events (Fig. 5). Meanwhile, 1 conventional PET image with 4 motion events was assessed as having visually poor image quality. The reader may recognize that the SUVR in brain PET cases with obvious large head movements is not reliable. However, the values obtained from PET cases with visually undetectable, small head movements may also be unreliable. In this study, the difference in UR between the conventional and motion-corrected PET images was significantly greater in the group with head motion than in the group without head motion. Therefore, head motion during brain PET imaging can affect image interpretation by making quantitative values inaccurate. This problem would be particularly severe in the evaluation of treatment responses by comparing PET images before and after treatment. In the group with head motion, the severity of the SUVR error can vary depending on the degree of amyloid deposition and the magnitude of motion, rather than the number of motions.

## Conclusion

The evaluation and correction of head motion in amyloid brain PET imaging is not merely supplementary information, as restricted movement can negatively affect both visual images and quantitative diagnostic assessments of the small structures of the brain. The data-driven algorithm presented herein provides a tool by which patient head motion incurred during PET imaging can be assessed and corrected post hoc, offering substantial advantages with respect to improving image quality and enhancing interobserver agreement. The proposed motion correction method also has the potential to be applied to brain PET scans with other molecular imaging probes.

## Data Availability

The datasets generated during and/or analyzed during the current study are available from the corresponding author on reasonable request.
